# Tips for an effective DR screening service in sub-Saharan Africa

**Published:** 2015

**Authors:** Tunde Peto

**Affiliations:** Head: Ophthalmic Image Analysis Unit, Moorfields Eye Hospital NHS Foundation Trust and UCL Institute of Ophthalmology, London, UK.

Screening for diabetic retinopathy is of increasing importance. It requires identification of people with diabetes, an accurate examination and/or photograph of the retina and competent analysis of the findings and/or images.

It is imperative that, where screening exists, treatment is available and that it is affordable and sustainable. Laser treatment is the mainstay of treatment in sub-Saharan Africa and it is important that laser machines are maintained and that an eye health professional is taught to provide safe and adequate laser treatment.

In addition to diabetic retinopathy, screening can pick up other ocular diseases, either as media opacities or abnormalities of the fundus. Fundal abnormalities include vascular diseases (e.g. arterial and venous occlusions), glaucomatous disc changes, age-related diseases, and some inherited retinal diseases.

Health care professionals working in the hospitals that receive patients from screening should be fully aware of how the screening is carried out, what the referral criteria are from screening to treatment and how and when they can discharge the patients back for follow-up. A good referral pathway will allow for growth (based on growing demand) and will build trust between health care providers and the population.

**Figure:**
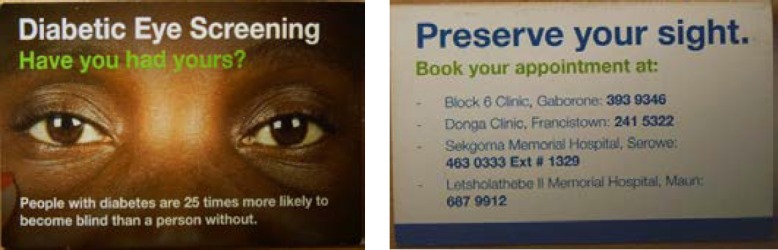


Business card-sized promotional materials are available at health centres and the Ministry of Health in Botswana. CREDIT: Peter Blow

